# Prediction for post-ERCP pancreatitis in non-elderly patients with common bile duct stones: a cross-sectional study at a major Chinese tertiary hospital (2015–2023)

**DOI:** 10.1186/s12911-024-02541-z

**Published:** 2024-05-28

**Authors:** Chaoqun Yan, Jinxin Zheng, Haizheng Tang, Changjian Fang, Jiang Zhu, Hu Feng, Hao Huang, Yilin Su, Gang Wang, Cheng Wang

**Affiliations:** 1 Department of Biliary and Pancreatic Surgery, Division of Life Sciences and Medicine, The First Affiliated Hospital of USTC, University of Science and Technology of China, No. 17 Lujiang Road, Hefei, 230001 Anhui Province China; 2 School of Global Health, Chinese Centre for Tropical Diseases Research, Shanghai Jiao Tong University School of Medicine, Shanghai, 200025 China; 3 Department of Pediatric Surgery, Division of Life Sciences and Medicine, The First Affiliated Hospital of USTC, University of Science and Technology of China, No. 17 Lujiang Road, Hefei, 230001 Anhui Province China; 4 Liver Transplantation Center, West China Hospital, Sichuan University, Chengdu, 610041 China

**Keywords:** Post-ERCP pancreatitis, ERCP, Common bile duct stones, Prediction

## Abstract

**Background:**

Post-ERCP pancreatitis is one of the most common adverse events in ERCP-related procedures. The purpose of this study is to construct an online model to predict the risk of post-ERCP pancreatitis in non-elderly patients with common bile duct stones through screening of relevant clinical parameters.

**Methods:**

A total of 919 cases were selected from 7154 cases from a major Chinese tertiary hospital. Multivariable logistic regression model was fitted using the variables selected by the LASSO regression from 28 potential predictor variables. The internal and external validation was assessed by evaluating the receiver operating characteristic curve and the area under curve. Restricted cubic spline modelling was used to explore non-linear associations. The interactive Web application developed for risk prediction was built using the R “shiny” package.

**Results:**

The incidence of post-ERCP pancreatitis was 5.22% (48/919) and significantly higher in non-elderly patients with female, high blood pressure, the history of pancreatitis, difficult intubation, endoscopic sphincterotomy, lower alkaline phosphatase and smaller diameter of common bile duct. The predictive performance in the test and external validation set was 0.915 (95% CI, 0.858–0.972) and 0.838 (95% CI, 0.689–0.986), respectively. The multivariate restricted cubic spline results showed that the incidence of pancreatitis was increased at 33–50 years old, neutrophil percentage > 58.90%, hemoglobin > 131 g/L, platelet < 203.04 or > 241.40 × 109/L, total bilirubin > 18.39 umol / L, aspartate amino transferase < 36.56 IU / L, alkaline phosphatase < 124.92 IU / L, Albumin < 42.21 g / L and common bile duct diameter between 7.25 and 10.02 mm. In addition, a web server was developed that supports query for immediate PEP risk.

**Conclusion:**

The visualized networked version of the above model is able to most accurately predict the risk of PEP in non-elderly patients with choledocholithiasis and allows clinicians to assess the risk of PEP in real time and provide preventive treatment measures as early as possible.

## Induction

Common bile duct stones (CBDS) are a relatively common chronic and recurrent digestive disease. The incidence of cholelithiasis is 5–15%, of which common bile duct stones account for 5–30% [1]. Common bile duct stones are divided into primary stones and secondary stones according to their source, and secondary common bile duct stones are the most common cause of common bile duct stones [2]. In recent years, the incidence of choledocholithiasis has been on the rise, with dietary factors and other factors changing [3].

Stone extraction by endoscopic retrograde cholangiopancreatography (ERCP) has become an ideal method for the treatment of common bile duct stones, which is safe and effective for asymptomatic and symptomatic patients, and has the advantages of less trauma, fast recovery and few complications [4]. The complications after ERCP-related procedures mainly include asymptomatic hyperamylaseemia, pancreatitis, cholangitis, cholecystitis, perforation, bleeding, etc., and even death in severe cases [4]. And post-ERCP pancreatitis (PEP) is one of the most common adverse events (AEs), and it has the potential to cause morbidity and mortality from clinical complications [5]. There are many risk factors for PEP, mainly divided into patient-related factors and ERCP procedure-related factors. Patient-related factors include sex, age, suspected oddi sphincter dysfunction (SOD), history of pancreatitis, and common bile duct diameter, and procedural factors include difficulty intubation and so on. Although many studies have detailed risk factors for the development of PEP, the factors reported vary widely due to different intubation techniques, heterogeneous patient populations, and included indicators [6, 7].

However, there is currently no established protocol to predict the risk of pancreatitis in non-elderly patients with common bile duct stones. In this study, we used the logistic regression model to explore the relationship between the clinical characteristics and PEP in non-elderly patients with CBDs to predict the risk of PEP. The model was translated to an online web that can be used by clinicians to determine the medical strategies for non-elderly patients with CBDs at high risk for PEP.

## Materials and methods

### Study design and patients

**Fig. 1:**
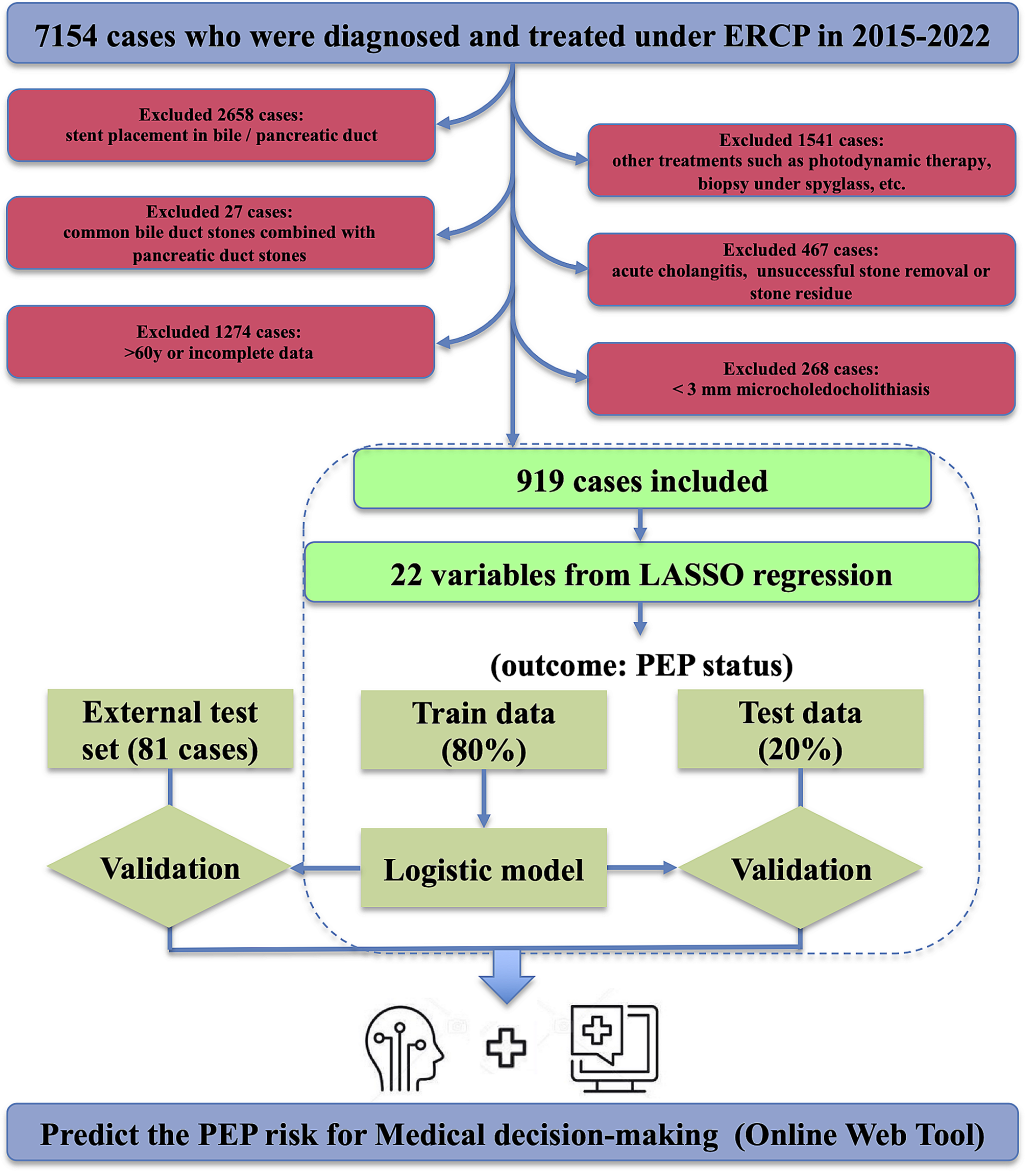
The workflow chart of the application of online model in the prediction of post-ERCP pancreatitis (PEP) in non-elderly patients with common bile duct stones

This was a retrospective, single-center study conducted at the First Affiliated Hospital of USTC. The research was approved by the ethics committee of the hospital (No. 2023-RE-207) and conducted in accordance with the guidelines of the Helsinki Declaration. During the periods between January 2015 and January 2023, 7154 consecutive ERCP procedures were performed. Of these 7154 cases, patients with bile duct or pancreatic duct stent placement, other treatments such as photodynamic therapy, biopsy under spyglass, etc., common bile duct stones combined with pancreatic duct stones, acute cholangitis, unsuccessful stone removal, residual stones, > 60 years old, incomplete data, a stone size of < 3 mm were excluded (Fig. 1). None of the patients were treated with rectal NSAIDs or hydration with lactated Ringer’s solution for regular use. Above all, 919 cases were included in our study. Since it was a retrospective analysis, the need for informed consent was waived. For external validation, the dataset comprised clinical data from random patients meeting the aforementioned criteria across multiple centers in Anhui Provincial, recorded from January 2021 to January 2023. The data came from the six centers of the First Affiliated Hospital of Anhui Medical University, Anhui No.2 Provincial People’s Hospital, the First Affiliated Hospital of Bengbu Medical College, the Second People’s Hospital of Hefei, the First Affiliated Hospital of Wannan Medical College, and the First People’s Hospital of Hefei, with a total of 81 cases.

### Study protocol and definitions

All ERCP procedures were conducted by the same operating and nursing team to avoid unnecessary bias. Briefly, using a catheter and guidewire, the common bile duct was cannulated under fluoroscopic guidance and performs an initial cholangiogram to delineate the anatomy by the operator. The stone removal procedure is then completed under fluoroscopy and guidewire guidance. Patient characteristics, including gender, age, BMI, HBP, DM, related medical history, surgical intervention, postoperative serology, and complications after ERCP were collected. The study endpoint was the risk prediction for the development of PEP. Difficult cannulation was defined as more than five cannulation attempts, a long cannulation time (> 5 min), or unintentional pancreatic duct cannulation occurring more than three times [8]. The definition of pancreatitis were based on the consensus criteria [9]. A diagnosis of PEP was given when patients had new-onset or worsened pancreatic-type abdominal pain lasted for at least 24 h after procedure, with an increased serum amylase level of more than three times higher than the normal upper limit.

### Selection and analysis of risk factors

Univariable analysis was performed to select 28 potential predictor variables for PEP based on previous related research. Multivariable logistic regression model was fitted using the variables selected by the LASSO regression. The λ value with the least binomial deviance was used for the final LASSO regression by conducting 10-fold cross validation method. Lower values on these fit statistics indicate better model fit. The association of each factor with the risk of PEP was estimated with odds ratios (ORs) and 95% CIs. Predictive power was assessed by evaluating the receiver operating characteristic (ROC) curve and the area under curve. Restricted cubic spline modelling was used to explore non-linear associations.

### Statistical analysis

All statistics were analyzed in R software (version 4.0.2; http://www.Rproject.org). The continuous variables were compared by a two-sided paired *t*-test. For categorical variables, the Person’s ${\chi }^{2}$ test was used to compare the frequency distributions between 2 groups.

### Online web application

After validation, the best performance model consisting of multiple clinical indicators was used as the web-based risk calculator. The interactive Web application developed for risk prediction of PEP in CBD Patients was built using the R “shiny” package. The related workflow chart was displayed in Fig. 1.

## Results

### Clinical demographics and characteristics of non-elderly patients with CBDs

The data of 919 non-elderly patients with CBDs was summarized, and the demographic and clinical characteristics are shown in Table 1. The mean age of all patients was 46.61 ± 10.63 years, and the female to male ratio in this subgroup was 1.30:1 (520 vs. 399), respectively. The prevalence of PEP was 5.22% (48/919). The incidence of PEP was significantly higher in non-elderly CBDs patients with female (77.08% vs. 55.45%, *p* = 0.003), HBP (29.17% vs. 16.88%, *p* = 0.029), the history of pancreatitis (20.83% vs. 5.28%, *p* = 0.000), difficult intubation (27.08% vs. 7.23%, *p* = 0.000), EST (58.33% vs. 39.72%, *p* = 0.011), lower ALP (136.85 ± 108.57 vs. 183.47 ± 153.40, *p* = 0.006) and smaller diameter of common bile duct (8.80 ± 2.79 vs. 10.21 ± 4.32, *p* = 0.002).

**Table 1:** Clinicopathologic characteristics of cohort in PEP patients with CBDs

Variables	All	Non-PEP (*n* = 871)	PEP (*n* = 48)	${\chi }^{2}$/t	*p*
Gender (Male / female)	399/520	388/483	11/37	8.664	0.003
Age (year)	46.61 ± 10.63	46.67 ± 10.71	47.19 ± 9.04	0.390	0.697
BMI (kg / m^2^)	23.33 ± 3.37	23.34 ± 3.38	23.17 ± 3.21	0.331	0.741
HBP (NO / YES)	758/161	724/147	34/14	4.755	0.029
DM (NO / YES)	851/68	805/66	46/2	0.355	0.551
History of pancreatitis (NO / YES)	863/56	825/46	38/10	16.607	0.000
History of hepatitis (NO / YES)	896/23	848/23	48/0	0.443	0.506
Presence of the gallbladder (NO / YES)	538/381	504/367	34/14	3.153	0.076
With gallstone (NO / YES)	650/269	611/260	39/9	2.708	0.100
With intrahepatic bile duct stones (NO / YES)	839/80	795/76	44/4	0.000	1.000
Number of Stones (1 / 2 / multiple)	491/69/359	462/67/342	29/2/17	1.067	0.572
Type of duodenal papillae (papillary / other)	878/41	832/39	46/2	0.000	1.000
Position relationship between papillae and diverticula (in / next / non- diverticula)	28/182/709	28/171/672	0/11/37	1.229	0.512
Difficult cannulation (NO / YES)	843/76	808/63	35/13	21.086	0.000
EST (NO / YES)	545/374	525/346	20/28	6.528	0.011
EPBD (NO / YES)	212/707	200/671	12/36	0.106	0.744
Hb (g / L)	130.39 ± 16.67	130.50 ± 16.77	128.33 ± 14.72	0.877	0.381
PLT (10^9^ / L)	59.63 ± 11.90	211.73 ± 74.25	204.33 ± 83.05	0.667	0.505
WBC (10^9^ / L)	5.83 ± 2.28	5.86 ± 2.29	5.23 ± 2.18	1.873	0.061
N (%)	211.34 ± 74.71	59.51 ± 11.92	61.76 ± 11.48	1.272	0.204
TB (umol / L)	35.12 ± 45.98	35.24 ± 45.83	32.99 ± 49.09	0.329	0.742
DB (umol / L)	20.90 ± 35.89	21.08 ± 35.78	17.78 ± 38.11	0.620	0.535
AST (IU / L)	94.74 ± 139.80	96.23 ± 141.60	67.61 ± 99.06	1.897	0.063
ALP (IU / L)	181.03 ± 151.70	183.47 ± 153.40	136.85 ± 108.57	2.824	0.006
GGT	321.20 ± 364.79	325.93 ± 367.12	235.33 ± 310.52	1.677	0.094
ALB (g / L)	42.23 ± 4.13	42.27 ± 4.14	41.45 ± 3.77	1.341	0.180
Diameter of common bile duct	10.14 ± 4.27	10.21 ± 4.32	8.80 ± 2.79	3.291	0.002
Diameter of stone	7.25 ± 3.21	7.29 ± 3.25	6.54 ± 2.20	1.565	0.118

### Screening of relevant clinical parametric variables

**Fig. 2:**
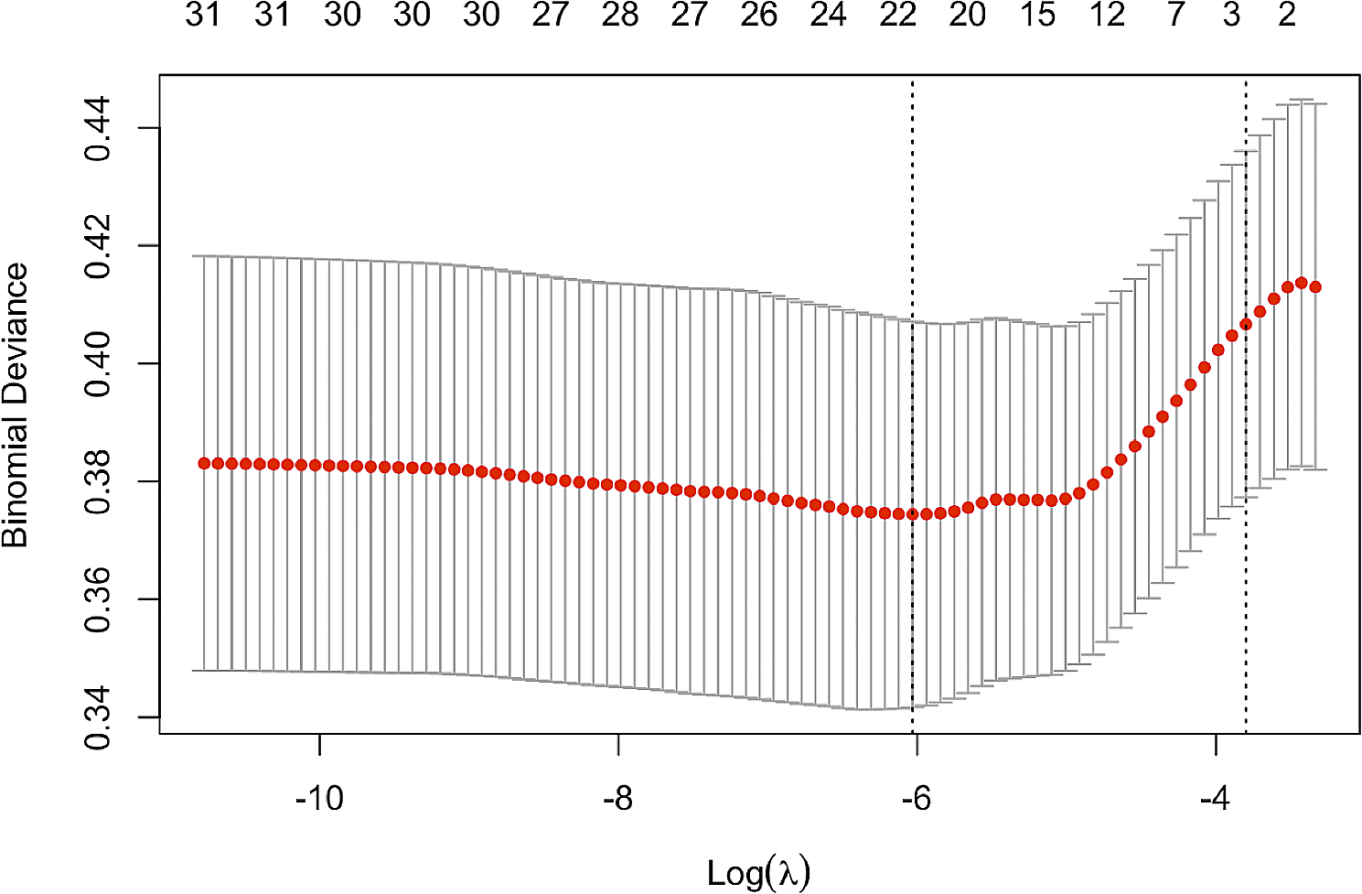
The screening of relevant variables was determined by lasso logistic regression

In order to potentially clinically used the risk model and predicted more precisely the occurrence of PEP. A total of 22 non-zero coefficients were selected from 28 variables in Table 1 by the lasso logistic regression model for the minimum Lambda value (0.002403). These 22 features are considered important for predicting the target variable (Fig. 2). The entire data set was partitioned into independent sets of training (80%; 736) and test (20%; 183) data, respectively, for model development and validation. Data were randomly sampled and repeated until no significant differences were observed in the above clinical variables between the two datasets (Table 2).

**Table 2:** Clinical data of training and validation data set in non-elderly patients with common bile duct stones

Variables	Training set (*N* = 736)	Validation set (*N* = 183)	${\chi }^{2}$/t	*p*
Gender (Male/ female)	323/413	76/107	0.331	0.565
HBP (NO / YES)	611/125	147/36	0.733	0.392
DM (NO / YES)	682/54	169/14	0.021	0.885
History of pancreatitis (NO / YES)	696/40	167/16	2.803	0.094
History of hepatitis (NO / YES)	717/19	179/4	0.002	0.966
Presence of the gallbladder (NO / YES)	427/309	111/72	0.421	0.517
With gallstone (NO / YES)	511/225	139/44	3.016	0.082
Number of Stones (1 / 2 / multiple)	383/58/295	108/11/64	2.997	0.223
Type of duodenal papillae (papillary / other)	703/33	175/8	0.004	0.948
Difficult cannulation (NO / YES)	675/61	168/15	0.002	0.968
EST (NO / YES)	436/300	109/74	0.006	0.936
EPBD (NO / YES)	173/563	39/144	0.398	0.528
Age (year)	46.67 ± 10.69	46.34 ± 10.37	0.371	0.711
Hb (g/L)	130.52 ± 16.45	129.87 ± 17.55	0.466	0.641
PLT (10^9^/L)	211.60 ± 75.87	210.30 ± 70.00	0.211	0.833
N (%)	59.58 ± 11.71	59.85 ± 12.67	0.277	0.781
TB (umol / L)	35.44 ± 45.90	33.85 ± 46.42	0.418	0.676
AST (IU / L)	92.11 ± 131.53	105.30 ± 169.03	0.984	0.326
ALP (IU / L)	178.70 ± 146.88	190.39 ± 169.83	0.933	0.351
ALB (g / L)	42.22 ± 4.12	42.28 ± 4.15	0.196	0.844
Diameter of common bile duct	10.11 ± 4.29	10.23 ± 4.20	0.346	0.730
Diameter of stone	7.23 ± 3.19	7.32 ± 3.28	0.354	0.724
PEP (NO / YES)	697/39	174/9	0.043	0.836

### Putative predictive factors and predictive model

**Fig. 3:**
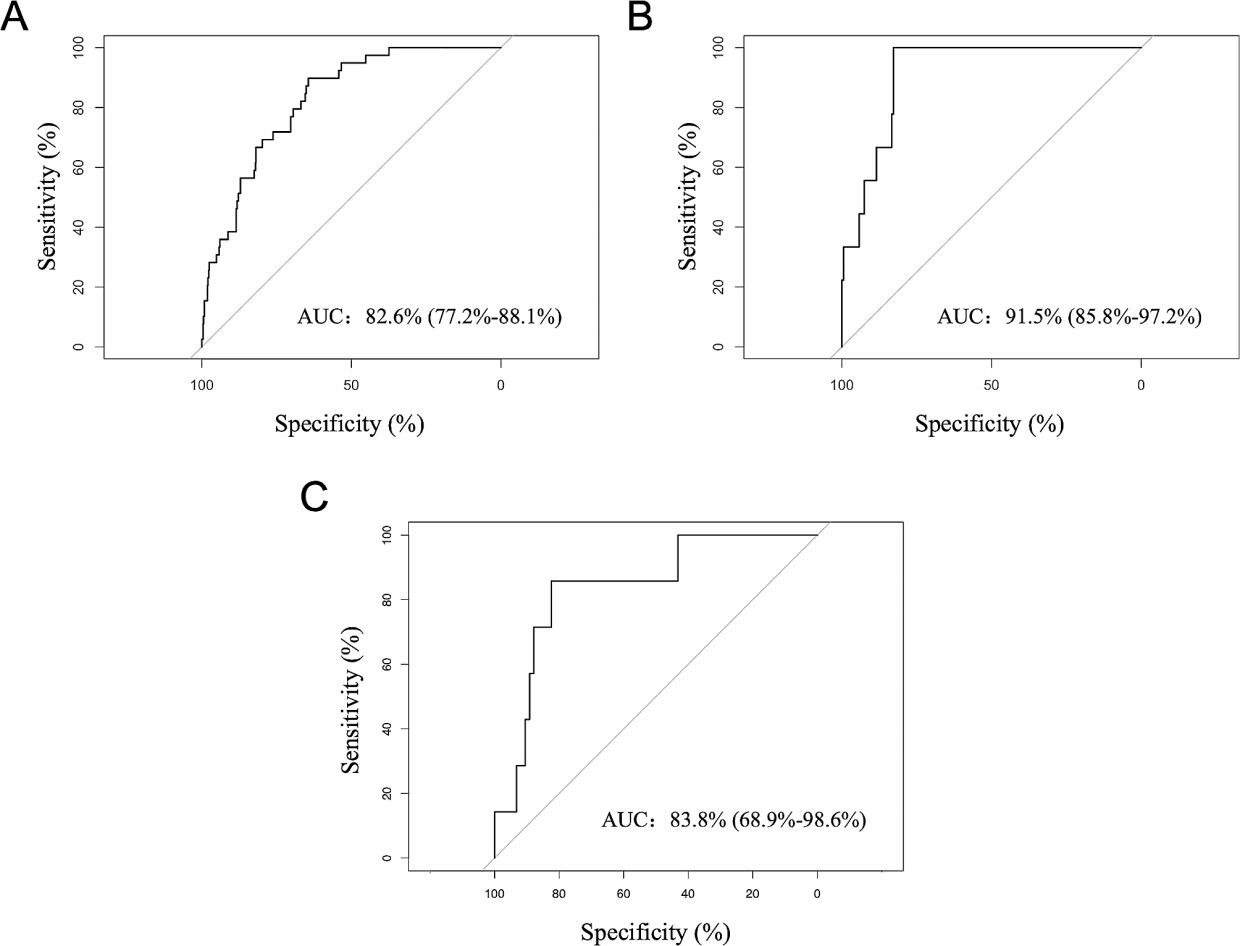
Receiver operating characteristic (ROC) curve plot in the train (**A**) and test (**B**) data; ROC curve plot in the external test set (**C**)

After maternal variables were adjusted, the risk of PEP was 4.66 (95% CI, 1.76–13.50; *p* = 0.003) times higher in female, 2.57 (95% CI, 1.02–6.24; *p* = 0.039) times higher in those with HBP, 6.07 (95% CI, 2.05–16.85; *p* = 0.001) times higher with pancreatitis history, 2.94 (95% CI, 1.20–7.17; *p* = 0.017) times higher if the EST was used (Table 3). We accessed the logistic regression model with the above 22 variables. As shown in the receiver operating characteristic curves (ROC), the train (Fig. 3A) and test (Fig. 3B) set exhibited strong predictive performance, with an area under the curve (AUC) of 0.826 (95% CI, 0.772–0.881) and 0.915 (95% CI, 0.858–0.972), respectively. In addition, the AUC was 0.838 (95% CI, 0.689–0.986) in the external validation set, as shown in Fig. 3C.

**Table 3:** Crude and adjusted analysis of PEP patients in the training set with categorical variables

Variables	PEP (*n*, %)	Crude analysis^a^	*p*	Adjusted analysis^b^	*p*
Female	30 (7.26)	2.73 (1.33–6.19)	0.009	4.66 (1.76–13.50)	0.003
HBP	10 (8.00)	1.75 (0.79–3.57)	0.144	2.57 (1.02–6.24)	0.039
DM	1 (1.85)	0.32 (0.02–1.52)	0.265	0.23 (0.01–1.32)	0.180
History of pancreatitis	7 (17.50)	4.40 (1.68–10.23)	0.001	6.07 (2.05–16.85)	0.001
History of hepatitis	0 (0.00)	0.00 (NA-8.24e16)	0.987	0.00 (0.00-1.70e)	0.987
Presence of the gallbladder	12 (3.88)	0.60 (0.29–1.17)	0.149	0.62 (0.16–1.85)	0.428
With gallstone	8 (3.56)	0.57 (0.24–1.20)	0.166	0.67 (0.18–2.74)	0.544
Number of Stones (2)	1 (1.72)	0.29 (0.02–1.41)	0.228	0.16 (0.01-1.00)	0.110
Number of Stones (multiple)	16 (5.42)	0.94 (0.48–1.82)	0.857	0.92 (0.42–1.94)	0.819
Type of duodenal papillae (other)	2 (6.06)	1.16 (0.18–4.05)	0.842	1.15 (0.17–4.69)	0.859
Difficult cannulation	8 (13.11)	3.14 (1.29–6.87)	0.007	2.36 (0.85–6.05)	0.084
EST	22 (7.33)	1.95 (1.02–3.79)	0.044	2.94 (1.20–7.17)	0.017
EPBD	30 (5.33)	1.03 (0.50–2.33)	0.948	2.27 (0.82–6.59)	0.119

^a^ Performed with a univariable logistic regression model. ^b^ Performed with a multivariable logistic regression model

### Univariable and multivariable restricted cubic spline function for PEP

**Fig. 4:**
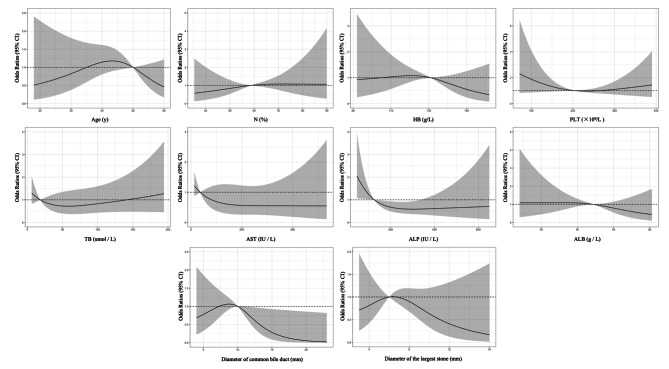
The restricted spline curve of univariable linear regression models were used to explore the potential relationship between relevant clinical indicators and PEP

**Fig. 5:**
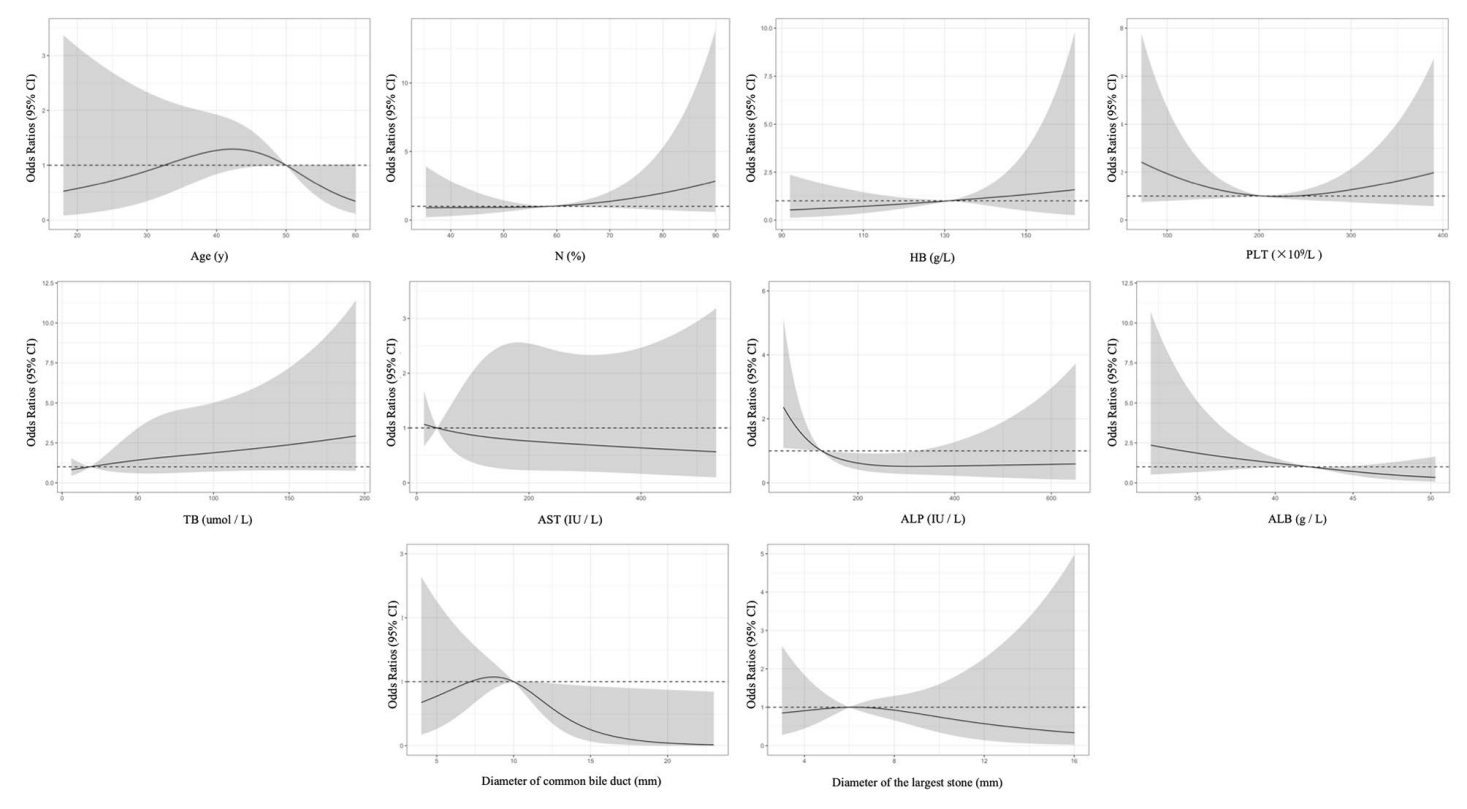
The restricted spline curve of multivariable linear regression models was used to explore the potential relationship between relevant clinical indicators and PEP

Restricted cubic spline (RCS) was used to clarify the relationship between relevant nonlinear clinical parameters and PEP. Univariate RCS results showed that the incidence of pancreatitis was increased in 35–50 years old, N% > 58.90%, HB 105.36–130.69 g/L, PLT < 204.64 or > 265.38 × 10^9^/L, TB > 141.27 or < 18.39 umol / L, AST < 36.56 IU / L, ALP < 124.92 IU / L, ALB < 42.21 g / L, and common bile duct diameter between 7.34 and 10.02 mm (Fig. 4). And the multivariate RCS results showed that the incidence of pancreatitis was increased at 33–50 years old, HB > 131 g/L, PLT < 203.04 or > 241.40 × 10^9^/L, TB > 18.39 umol / L, and common bile duct diameter between 7.25 and 10.02 mm. It is worth mentioning that the ranges of neutrophils, AST, ALP, and ALB in the corrected multivariate RCS results are the same as in the univariate results (Fig. 5).

### Online web application

**Fig. 6:**
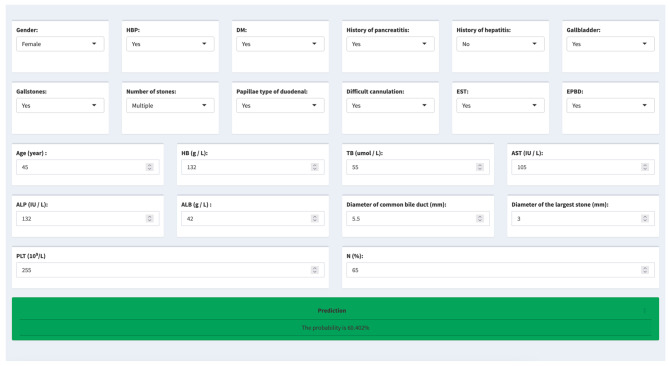
The web server was developed for immediate PEP risk by 22 features

The regression model based on the coefficients of each variable was used to create a web-based risk prediction calculator. A web server based on the Shiny application in R was developed that supports query for immediate PEP risk: https://jamesjin63.shinyapps.io/Shiny_PEP/. The related flow chart of the work was shown in Fig. 6.

## Discussion

As an invasive operation, ERCP is diverse, complex and difficult, and complications such as PEP, bleeding, perforation, and postoperative reinfection are important factors affecting the outcome and prognosis of ERCP [10]. The overall incidence of all postoperative complications of ERCP worldwide is 6–15%, with PEP remaining the most common complication [11]. A 2023 meta-analysis of PEP morbidity and mortality based on 145 RCTs (*n* = 19,038) showed an overall incidence of PEP of 10.2% and an incidence of 14.1% in high-risk patients [12]. In this study, as one of the first centers in China to carry out ERCP-related procedures, our incidence of PEP was 5.22%, which was lower than the overall incidence worldwide. Mild PEP may lead to clinical symptoms such as mild abdominal pain and prolonged hospital stay, while severe cases may lead to pancreatic edema, pancreatic necrosis, systemic inflammatory response syndrome (SIRS), multiple organ failure, and even death [13]. Therefore, identifying risk factors for the development of PEP and early intervention and treatment are key to reducing morbidity and mortality.

The risk factors for PEP that have been recognized by the existing results have been mentioned above, but there are still differences in the underlying disease, related medical history, preoperative serology, anatomical relationships and so on. Multiple risk factors for PEP have a synergistic effect when co-existing, rather than a simple probability, and the incidence of PEP can be as high as 30–50% when multiple risk factors are combined [14].

Previous studies have shown that younger patients are more likely to complicate PEP, but cut-off values vary between studies, mostly 50–60 years old or younger [15, 16]. Pancreatic exocrine function increases linearly before age 43 and begins to decline gradually after age 43, resulting in reduced response to mechanical injury stimuli such as ERCP [17]. Based on this, this study excluded patients over 60 years of age to further investigate the risk factors for PEP in non-elderly patients, and the multivariate RCS results showed that patients aged 33–50 years had a higher risk of developing PEP. This information is reported for the first time for the age range of high risk of PEP in non-eldly patients.

Female, the history of pancreatitis, difficult intubation and EST in this study are all risk factors for PEP, which have been reported in multiple studies [18–20]. Interestingly, we found that patients with HBP were more likely to develop PEP in this study. The main reason for considering is that most hypertensive patients have abnormal lipid metabolism, and patients with hyperlipidaemia had a significantly increased risk of PEP [21]. It has been reported that common bile duct less than 10 mm is a risk factor for PEP [22], which is basically consistent with the high-risk of PEP in patients with a diameter of common bile duct between 7.25 and 10.02 mm in this study. The diameter of the common bile duct is an indicator of biliary obstruction, and the increase in the diameter indicates that there is obstruction at the end of the biliary tract, and the widened common bile duct relieves the pressure in the bile duct and pancreatic duct to some extent, thereby reducing the return of bile or pancreatic juice and reducing the chance of PEP. To date, preoperative serology of patients has not been included as a quantitative variable in the prediction of PEP. Our multivariate RCS results showed that HB > 131 g/L, PLT < 203.04 or > 241.40 × 109/L, TB > 18.39 umol/L, AST < 36.56 IU/L, ALP < 124.92 IU/L, ALB < 42.21 g/L all increased the risk of PEP. Risk stratification based on their serologic status and PEP would be the next step.

To summarize, many clinical parameters set as prediction variables are already known to favor PEP in clinical, but these parameters are not independent. Therefore, we established a simple website-based risk prediction tool using demographic and clinical variables to evaluate the probability of PEP in non-eldly patients with CBDs, aid in clinical decision making, and ultimately improve more patients to benefit from the model. Our purpose is to provide real-time prediction of PEP incidence to clinicians clearly and easily so that clinicians can administer preventive treatment of PEP as early as possible through our visualization webpage.

This study confirmed that it has good predictive performance according to internal and external verification and can find high-risk groups of PEP according to relevant data, and give preventive measures and treatment plans before PEP, thereby slowing down the progression of PEP. Limitations are inevitable in retrospective, observational studies and we need prospective, multicenter studies with larger sample sizes to validate the external applicability of this model.

In conclusion, the incidence of post-ERCP pancreatitis was 5.22% (48/919) and significantly higher in non-elderly patients with female, HBP, the history of pancreatitis, difficult intubation, EST, lower ALP and smaller diameter of common bile duct. And we screened 22 non-zero coefficients from 28 relevant variables and constructed a regression model to predict the risk of PEP in non-elderly patients with CBDs with high accuracy by using internal and external model validation. The predictive performance in the test data and external test set was 0.915 and 0.838, respectively. We adopted an innovative approach to clarify the relationship between age, HB, TB, AST, diameter of common bile duct and other related nonlinear clinical parameters and PEP, and further refined the numerical intervals of high PEP risk by multivariate RCS. A visualized web version will allow clinicians to assess the risk of PEP in patients with CBDs in real time based on the above results and provide preventive treatment measures as early as possible.

## Data Availability

The datasets used and/or analysed during the current study are available from the corresponding author on reasonable request.

## Abbreviations

AEs: Adverse events
ALB: Albumin
ALP: Alkaline phosphatase
AST: Aspartate amino transferase
AUC: Area under the curve
BMI: Body Mass Index
CBDS: Common bile duct stones
CI: Confidence interval
DM: diabetes mellitus
ERCP: Endoscopic retrograde cholangiopancreatography
EST: Endoscopic Sphincterotomy
HB: Hemoglobin
HBP: High Blood Pressure
LASSO: Least absolute shrinkage and selection operator
N: Neutrophil
ORs: Odds ratios
PEP: Post-ERCP pancreatitis
PLT: Blood platelet
RCS: Restricted cubic spline
RCTs: Randomizedcontrolledtrial
ROC: receiver operating characteristic
SIRS: Systemic inflammatory response syndrome
SOD: Sphincter of Oddi dysfunction
TB: Total bilirubin
